# Subcutaneous emphysema as the first relevant clinical sign of complicated tubercular lymph node disease in a child

**DOI:** 10.1186/1471-2334-13-461

**Published:** 2013-10-04

**Authors:** Susanna Esposito, Alberto Giannini, Pietro Biondetti, Nicola Bonelli, Mario Nosotti, Samantha Bosis, Edoardo Calderini, Nicola Principi

**Affiliations:** 1 Pediatric Clinic 1, Department of Pathophysiology and Transplantation, Università degli Studi di Milano, Fondazione IRCCS Ca’ Granda Ospedale Maggiore Policlinico, Via Commenda 9, 20122 Milan, Italy; 2 Pediatric Intensive Care Unit, Fondazione IRCCS Ca’ Granda Ospedale Maggiore Policlinico, Milan, Italy; 3 Pediatric Radiology Unit, Fondazione IRCCS Ca’ Granda Ospedale Maggiore Policlinico, Milan, Italy; 4 Thoracic Surgery Unit, Department of Pathophysiology and Transplantation, Università degli Studi di Milano, Fondazione IRCCS Ca’ Granda Ospedale Maggiore Policlinico, Milan, Italy

**Keywords:** Children, *Mycobacterium tuberculosis*, Subcutaneous emphysema, Tuberculosis

## Abstract

**Background:**

Children make up a significant proportion of the global tuberculosis (TB) caseload, and experience considerable TB-related morbidity and mortality. Unfortunately, it is not easy to diagnose TB in the first years of life because of the diversity of its clinical presentation and the non-specific nature of most of its symptoms.

**Case presentation:**

A 26-month-old male child was admitted to hospital because of the sudden onset of rapidly increasing swelling of the neck, face and upper trunk a few hours before. Upon admission, his temperature was 36.5°C, pulse rate 120/min, respiratory rate 36/min, and O_2_ saturation 97% in air. Palpation revealed subcutaneous emphysema (SE) over the swollen skin areas, and an examination of the respiratory system revealed crepitations in the left part of the chest without any significant suggestion of mediastinal shift. Chest radiography showed enlargement of the left lung hilum with pneumomediastinum and diffuse SE. Bronchoscopy was carried out because of the suspicion that the SE may have been due to the inhalation of a peanut. This excluded the presence of a foreign body but showed that the left main bronchus was partially obstructed with caseous material and showed significant signs of granulomatous inflammation on the wall. Contrast-enhanced computed tomography of the lungs confirmed the SE and pneumomediastinum, and revealed bilateral hilum lymph node disease with infiltration of the adjacent anatomical structure and a considerable breach in the left primary bronchus wall conditioning the passage of air in the mediastinum and subcutaneous tissue. As a tuberculin skin test and polymerase chain reaction for *Mycobacterium tuberculosis* on bronchial material and gastric aspirate were positive, a diagnosis of TB was made and oral anti-TB therapy was started, which led to the elimination of *M. tuberculosis* and a positive clinical outcome.

**Conclusions:**

This is the first case in which SE was the first relevant clinical manifestation of TB and arose from infiltration of the bronchial wall secondary to caseous necrosis of the hilum lymph nodes. Physicians should be aware of the fact that SE is one of the possible initial signs and symptoms of early TB infection, and act accordingly.

## Background

In the industrialised countries such as Italy, the incidence of tuberculosis (TB) is relatively low and it is prevalent among specific populations, with the groups at highest risk being immigrants from highly endemic areas, drug addicts, ethnic minorities, refugees, the homeless and immunocompromised subjects [1]. However, infection has also been documented in low-risk populations, including a recent outbreak at a primary school in Milan that involved 15 school-children with active TB disease and 173 with latent infection, and the spread of infection in a maternity ward in Rome [2,3]. Disease control has been largely complicated by the emergence of multi-resistant *Mycobacterium tuberculosis* strains [4], which explains why greater attention is now being given to the diagnosis and treatment of TB in all countries, including those with very efficient health system [5].

Children make up a significant proportion of the global TB caseload, and experience considerable TB-related morbidity and mortality [6]. Unfortunately, it is not easy to diagnose TB in the first years of life because of the diversity of its clinical presentation and the non-specific nature of most of its symptoms [7,8]. The most frequent of these are a failure to thrive and reduced playfulness together with low-grade or intermittent fever, although cough and wheezing unresponsive to conventional treatment are common when the respiratory tract is extensively involved. The signs of disease are frequently subtle, which may delay the diagnosis and consequently increase the risk of progression to a significantly more devastating form. The appearance of subcutaneous emphysema (SE) as the first sign of disease is very rare.

We here describe the case of a child in whom SE was diagnosed before the presence of TB was suspected and subsequently demonstrated.

### Case presentation

A 26-month-old male child was admitted to the Pediatric Intensive Care Unit of the Fondazione IRRCS Ca’ Granda Ospedale Maggiore Policlinico, Milan, Italy, because of the sudden onset of rapidly increasing swelling of the neck, face and upper trunk a few hours before. The child’s medical history had been uneventful except for the onset of a non-productive cough two months previously and nightly intermittent mild fever for the previous 15 days. The administration of oral amoxicillin 50 mg/kg/day for 10 days had had no effect on the cough or fever. Furthermore, two days before admission, the child had ingested several peanuts before being noticed by his parents.

Upon admission, the patient was conscious and oriented: his weight was 13 kg, body temperature 36.5°C, pulse rate 120/min, respiratory rate 36/min, blood pressure 98/62 mm/Hg, and O_2_ saturation 97% in air. Palpation revealed SE over the swollen skin areas, and an examination of the respiratory system revealed crepitations in the left part of the chest without any significant suggestion of mediastinal shift. The results of cardiovascular and central nervous system examinations were normal.

**Figure 1:**
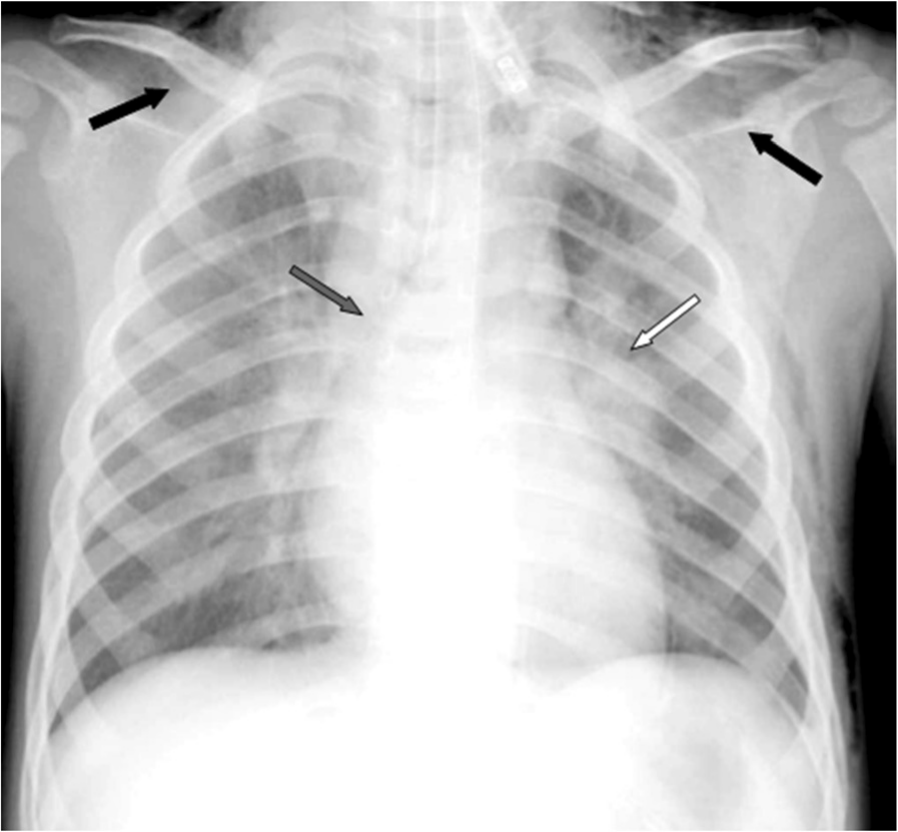
Chest X-ray showing a significant enlargement of the left lung hilum (white arrow) with pneumomediastinum (grey arrow) and subcutaneous emphysema (SE, black arrows).

Blood tests revealed normal hemoglobin with moderate leukocytosis (total leukocytes 15,300 mm^3^; differential count: neutrophils 61%, lymphocytes 30%). His C-reactive protein level was 7 mg/L. Arterial blood gas analysis showed pH 7.41, pCO_2_ 42 mm Hg and pO_2_ 90 mmHg. Chest radiography revealed significant enlargement of the left lung hilum with pneumomediastinum and SE (Figure 1).

**Figure 2:**
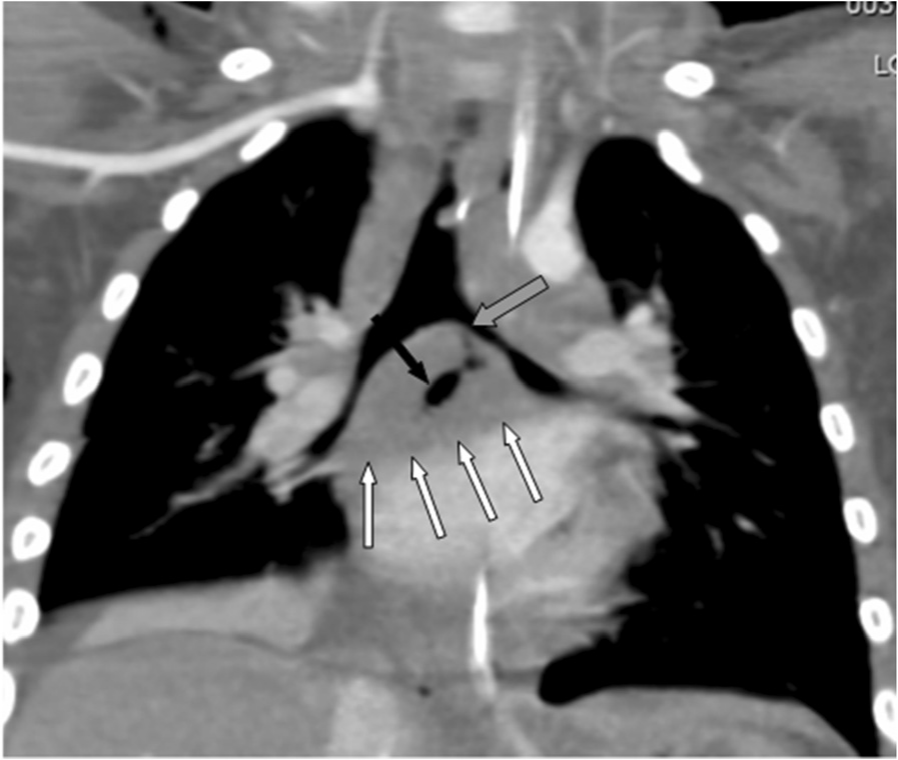
Coronal multiplanar reconstruction (MPR) showing the subcarinal and bilateral hilar lymphoadenopathy (white arrows), the fistulous tract (black arrow), and the breach in the left primary bronchus (grey arrow).

Flexible optical fibre bronchoscopy was carried out because of the suspicion the child may have inhaled a peanut. This excluded the presence of a foreign body, but showed that the left main bronchus was partially obstructed with caseous material and that there were significant signs of granulomatous inflammation on the wall. This material was extracted and sent to the laboratory for histological and microbiological examination. Contrast- enhanced computed tomography (CECT) of the lung confirmed SE and pneumomediastinum, and revealed bilateral hilum lymph node disease with infiltration of the adjacent anatomical structure, and a substantial breach in the left primary bronchus wall conditioning the passage of air in the mediastinum and subcutaneous tissue (Figure 2).

As a tuberculin skin test and polymerase chain reaction (PCR) for *M. tuberculosis* on bronchial material and gastric aspirate were positive, a diagnosis of TB was made and it was discovered that the child’s mother had a cavitary pulmonary TB.

An oral therapy with isoniazid (15 mg/kg/day), rifampin (16 mg/kg/day), pyrazinamide (40 mg/kg/day) and ethambutol (23 mg/kg/day) was started. The SE disappeared about one week after admission, and a control examination after 10 days of treatment revealed a significant increase in alanine aminotransferase serum levels (478 IU/L). This led to the discontinuation of rifampin after 2 weeks from the beginning of therapy and the dose of isoniazid was reduced to 10 mg/kg/day. Moreover, due to concerns about drug resistance (although when the culture became available *M. tuberculosis* resulted fully sensitive), amikacin (15 mg/kg/day) was added. Corticosteroids were not added to the treatment. A gastric aspirate evaluation four weeks after the beginning of therapy revealed that the treatment had eliminated *M. tuberculosis*. Amikacin was discontinued, the child was discharged and it was suggested to continue a 3-drug regimen for a total of two months followed by a 2-drug regimen for seven months, while returning periodically for further controls.

## Conclusions

SE and pneumomediastinum are relatively common in critically ill patients affected by blunt or penetrating trauma, soft-tissue infections, or any condition that creates a gradient between intra-alveolar and perivascular interstitial pressures (as in the case of inappropriate mechanical ventilation or foreign body inhalation) [9]. In children, they can occur at birth, or as a consequence of violent cough or straining at defecation, or during a severe asthma attack (particularly in subjects treated with inhaled bronchodilators and nebulisation) [10], or during the course of infectious diseases such as staphylococcal pneumonia [11], measles [12], *Pneumocystis jiroveci* pneumonia [13], influenza [14], bronchiolitis [15] or pertussis [16].

The occurrence of SE in patients with TB is rare and, in children, it has only been described in patients with miliary TB [17-20] and in one case in which lung involvement was secondary to rib TB [21]. To the best of our knowledge, ours is the first case in which SE was the first relevant clinical manifestation of TB and arose from infiltration of the bronchial wall secondary to caseous necrosis of the hilum lymph nodes. It is therefore easy to understand why the first suspicion was the inhalation of a foreign body, and TB was only considered when complicated lymph node disease was demonstrated by CT and confirmed by the evaluation of gastric aspirate and bronchial material.

Complicated lymph node disease is a clinical syndrome that could be one of the early manifestations of TB disease, usually occurring 4–12 months after primary infection. It is due to an excessive lymph node response with inadequate innate or adaptive immunity, or both. The most frequent clinical manifestations usually associated with a substantial deterioration in general health are wheezing and/or reduced ventilation and radiological changes include hyperinflation, atelectasis, lung collapse, and the expanding consolidation of a segment or entire lobe [22-24]. Tracheoesophageal or bronchoesophageal fistula are very rare presentations. With the exception of cough and mild intermittent fever, our patient showed no sign or symptom of disease, and his laboratory data were normal, including C-reactive protein levels. Chest radiography did not reveal any significant alterations, and only lung CT made it possible to suggest the diagnosis.

These findings highlight the exceptional nature of the case and underline the possible difficulties of promptly suspecting TB. Pediatricians should be aware that children presenting with SE may have TB and include this in their differential diagnosis.

### Consent

Written informed consent was obtained from the patient’s parents for publication of this Case report and any accompanying images. A copy of the written consent is available for review by the Editor of this journal.

## Abbreviations

CECT: Contrast-enhanced computed tomography; MPR: Multiplanar reconstruction; PCR: Polymerase chain reaction; SE: Subcutaneous emphysema; TB: Tuberculosis.

## Competing interests

The authors declare that they have no competing interests.

## Authors’ contributions

SE drafted the manuscript, supervised the management of TB, and was head of the pediatric clinic in which the patient was hospitalised; AG admitted the patient to the pediatric intensive care unit; PB and NB evaluated all of the chest radiographs and CT scan of the child; MN performed the bronchoscopy; SB followed the patient during hospitalisation and follow up; EC supervised the management of the patient in the pediatric intensive care unit; NP co-wrote the draft manuscript. All authors read and approved the final manuscript.

## Pre-publication history

The pre-publication history for this paper can be accessed here:

http://www.biomedcentral.com/1471-2334/13/461/prepub
